# *N*-Heterocyclic carbene-catalyzed direct cross-aza-benzoin reaction: Efficient synthesis of α-amino-β-keto esters

**DOI:** 10.3762/bjoc.8.169

**Published:** 2012-09-10

**Authors:** Takuya Uno, Yusuke Kobayashi, Yoshiji Takemoto

**Affiliations:** 1 Graduate School of Pharmaceutical Sciences, Kyoto University, Yoshida, Sakyo-ku, Kyoto 606-8501, Japan

**Keywords:** α-amino-β-keto esters, cross-aza-benzoin, α-imino ester, *N*-heterocyclic carbenes, organocatalysis, umpolung reactions

## Abstract

An efficient catalytic synthesis of α-amino-β-keto esters has been newly developed. Cross-coupling of various aldehydes with α-imino ester, catalyzed by *N*-heterocyclic carbene, leads chemoselectively to α-amino-β-keto esters in moderate to good yields with high atom efficiency. The reaction mechanism is discussed, and it is proposed that the α-amino-β-keto esters are formed under thermodynamic control.

## Introduction

**Figure 1:**
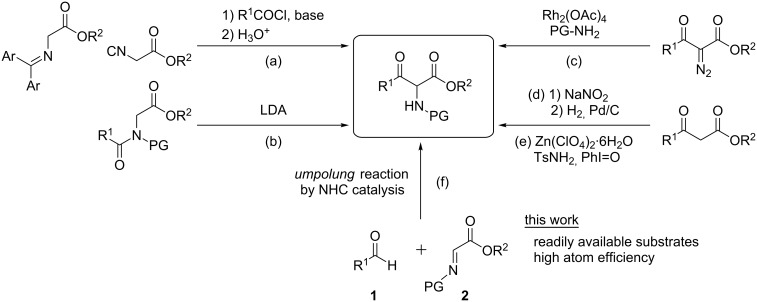
Synthetic methods for α-amino-β-keto esters.

α-Amino-β-keto ester derivatives are one of the fundamental structural subunits in natural products such as miuraenamides [1], and important building blocks for the synthesis of a variety of heterocyclic compounds [2] and pharmaceutically active products [3–5]. In addition, they are valuable intermediates for chiral α-amino acids [6–7], including β-hydroxy-α-amino acids [8–14]. Consequently, significant efforts have been devoted to synthesizing the privileged structure (Figure 1), and two main distinct approaches based on the bond-forming position have been developed. One approach utilizes C_α_–C_β_ bond formations, such as (a) acylation of Schiff bases or α-isocyano esters with acyl halides [4,15–17], and (b) intramolecular N–C acyl migration of the *N*-acyl glycine derivatives [18]. The other consists of C–N bond-forming reactions, such as (c) a rhodium-catalyzed N–H insertion reaction with α-diazo-β-keto esters [19–21], and (d) α-oxidation of β-keto esters to the corresponding oximes and the subsequent hydrogenation [22]. However, the former methods require a stoichiometric amount of strong bases, and the latter employ inaccessible substrates or multistep protocols. Recently, Zhang and co-workers reported Zn(ClO_4_)_2_·6H_2_O-catalyzed, mild and direct α-amination of β-keto esters with TsNH_2_, but in this case, a stoichiometric amount of PhI=O is needed as the oxidant (Figure 1, (e)) [23]. Therefore, mild, efficient and environmentally friendly strategies for the synthesis of these esters are still needed. We envisioned that highly atom-efficient synthesis of α-amino-β-keto esters could be achieved by a novel umpolung approach including C–C bond construction, that is, formal addition of acyl anion equivalents generated from aldehydes **1** with NHCs, to α-imino esters **2** (Figure 1, (f)).

Although a large number of NHC-catalyzed umpolung reactions, such as the benzoin reaction and Stetter reaction, have been developed [24–34], the related reactions of aldehydes with imines, i.e., the cross-aza-benzoin reactions, have been much less studied, in spite of the pharmaceutical and biological importance of providing α-amino ketones [35–43]. In particular, a reaction employing the imines directly has rarely been developed [40,42], due to the unproductive reaction of NHCs with the imines. Therefore, in situ generation of imines or iminium ions from their precursors is generally required to control their reactivity. We anticipated that the introduction of appropriate protecting groups of the nitrogen atom of α-imino esters **2**, which have been known to be excellent electrophiles, would suppress an unexpected reaction of **2** with NHCs, enabling the direct use of **2** as acyl anion acceptors in the cross-aza-benzoin reaction. In this communication, we describe a new, efficient, and atom-economical synthesis of the α-amino-β-keto esters by NHC-catalyzed cross-aza-benzoin reaction of aldehydes with α-imino esters under mild basic conditions.

## Results and Discussion

**Figure 2:**
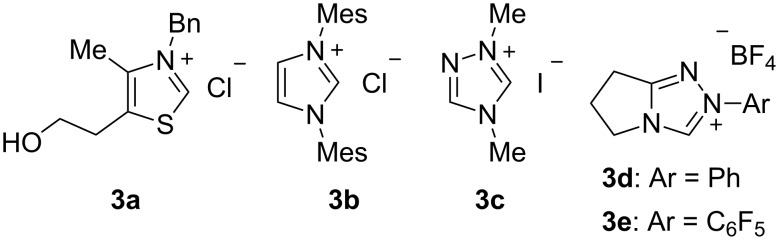
Structures of several NHC precatalysts.

First we initiated the cross-aza-benzoin reaction of benzaldehyde (**1a**) by employing 20 mol % of commercially available precatalyst **3a** (Figure 2) and K_2_CO_3_. Gratifyingly, when ethyl *N*-PMP-2-iminoacetate (**4**) was used as acyl anion acceptor [44], the reaction proceeded smoothly in THF at room temperature to generate the desired product **5a** in 58% yield (Table 1, entry 1). Surprisingly, no benzoin **6** arising from homo-coupling of **1a** was obtained. Encouraged by this result, we then attempted the other precatalysts **3b–e** depicted in Figure 2. Imidazolium salt **3b** and simple triazolium salt **3c** gave no coupled product **5a** (Table 1, entries 2 and 3). Further screening revealed that bicyclic triazolium salt **3d** could catalyze the reaction to give **5a** in 42% yield (Table 1, entry 4). We reasoned that the acidity of the NHC precursor was important to promote the reaction under mild basic conditions (Table 1, entry 3 versus entry 4), and therefore we next investigated the substituent on the nitrogen atom of NHC. As envisaged, *N*-pentafluorophenyl-substituted precatalyst **3e** [45–49], whose C-2 proton is more acidic than **3d**, furnished the coupled product **5a** in good yield (Table 1, entry 5). Subsequently, we attempted several bases to find that cesium carbonate gave slightly lower yield (Table 1, entry 6), whereas an amine base and a stronger base were not effective, due to the competitive decomposition of **4** under these conditions (Table 1, entries 7 and 8). Different solvents, such as CH_2_Cl_2_, toluene, and MeCN, have been tested in this reaction, and THF was found to be optimal in terms of chemical yield (Table 1, entries 9–11).

**Table 1:** Reaction optimization.^a^

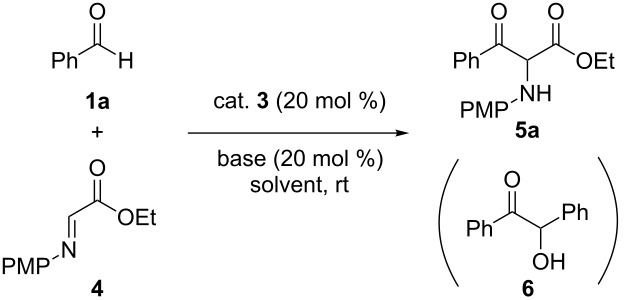				

Entry	Cat. **3**	Base	Solvent	Yield (%)^b^

1	**3a**	K_2_CO_3_	THF	58
2	**3b**	K_2_CO_3_	THF	<5
3	**3c**	K_2_CO_3_	THF	<5
4	**3d**	K_2_CO_3_	THF	42
5	**3e**	K_2_CO_3_	THF	70
6	**3e**	Cs_2_CO_3_	THF	66
7	**3e**	NEt_3_	THF	56
8	**3e**	KO*t*-Bu	THF	37
9	**3e**	K_2_CO_3_	CH_2_Cl_2_	61
10	**3e**	K_2_CO_3_	toluene	56
11	**3e**	K_2_CO_3_	MeCN	40

^a^Reactions conducted with **1a** (0.3 mmol) and **4** (1.3 equiv) in THF (0.5 M). ^b^Isolated yields.

With the efficient catalytic system in hand (20 mol % of **3e** and K_2_CO_3_ in THF at room temperature), we next evaluated the substrate generality with a variety of aromatic aldehydes **1** bearing a diverse range of functionality (Table 2). Chloro-, nitro-, cyano-, and methoxycarbonyl-substituted aromatic aldehydes were compatible with the reaction conditions (Table 2, entries 1–6). In all cases, the reaction led to the desired products in moderate to good yields. In addition, *ortho*-substituted aldehyde, which is considered to be a poor acyl donor in benzoin reactions [46], was also smoothly converted to the expected product **5d** in 61% yield (Table 2, entry 3). It is noteworthy that chloro-substituted aldehydes were tolerated, because the corresponding products **5b**–**d** could in principle undergo further functionalization by palladium-catalyzed cross-coupling reactions. Additionally, electron-rich aromatic aldehydes, which are often known to be less reactive in NHC-catalyzed reactions, could be coupled with **4** to provide **5h** and **5i** in 66% and 68% yields, respectively (Table 2, entries 7 and 8). Notably, heteroaromatic aldehydes, such as 3-thiophenecarboxaldehyde and furfural, were also successful in yielding the expected products **5j** and **5k** in 72% and 49% yield, respectively (Table 2, entries 9 and 10).

**Table 2:** Substrate scope.^a^

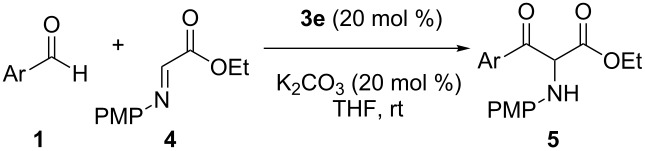			

Entry	Ar	**5**	Yield (%)^b^

1	4-ClC_6_H_4_	**5b**	69
2	3-ClC_6_H_4_	**5c**	59
3	2-ClC_6_H_4_	**5d**	61
4	4-NO_2_C_6_H_4_	**5e**	45
5	4-CNC_6_H_4_	**5f**	39
6	4-MeOCOC_6_H_4_	**5g**	73
7	4-MeC_6_H_4_	**5h**	66
8	4-MeOC_6_H_4_	**5i**	68
9	thiophen-3-yl	**5j**	72
10	furan-2-yl	**5k**	49

^a^Reactions performed with **1** (0.3 mmol) and **4** (1.3 equiv) in THF (0.5 M). ^b^Isolated yields.

**Scheme 1:**
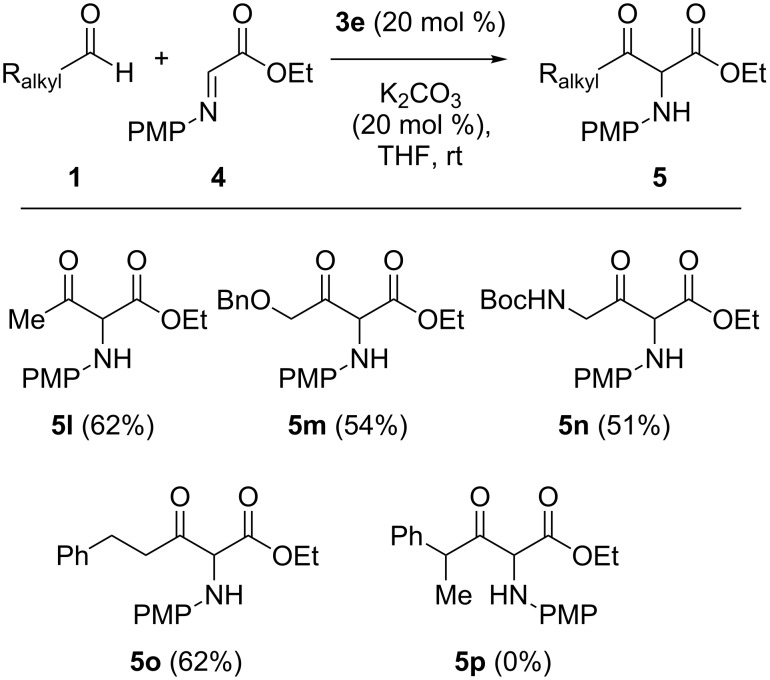
Scope of aliphatic aldehydes.

We next investigated the reaction with several aliphatic aldehydes (Scheme 1). Generally, unactivated aliphatic aldehydes are unsuccessful for the NHC-catalyzed reactions, because of their low electrophilicity relative to aromatic aldehydes [40,46–47,50]. To our delight, this methodology was found to be also suitable for the enolizable aliphatic aldehydes. Under the optimal reaction conditions (Table 1, entry 5), acetaldehyde and other primary alkyl aldehydes bearing functional groups such as ether, carbamate, and phenyl groups, were converted into the corresponding products **5l**–**o** in good yields. The α-branched aldehyde, however, failed to give the desired product **5p**, presumably due to the increased steric hindrance along with the inherently low electrophilicity.

**Scheme 2:**
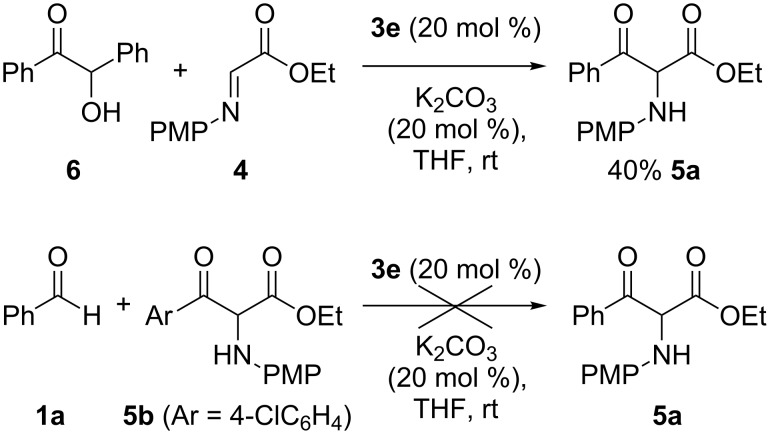
Cross-over experiments.

In order to elucidate the reaction mechanism, a 0.5 equiv of benzoin (**6**) was used instead of aldehyde **1a** under standard conditions, affording the cross-coupled product **5a** in 40% yield (Scheme 2). In addition, when the reaction was conducted with α-amino-β-keto ester **5b** in place of α-imino ester **4**, the cross-over product **5a** was not detected. These findings indicate that the formation of benzoin is reversible via the Breslow intermediate, whereas the retro-benzoin reaction of cross-coupled product **5** does not occur under the present reaction conditions, and that the product **5** is formed under thermodynamic control.

**Scheme 3:**
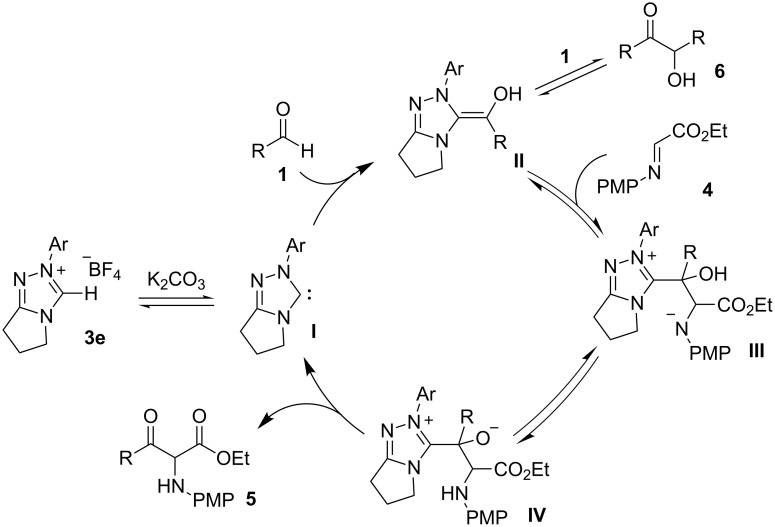
Proposed reaction mechanism.

A plausible mechanism for the cross-aza-benzoin reaction is shown in Scheme 3. Carbene **I** is generated by deprotonation of triazolium salt **3e** in the presence of K_2_CO_3_. The carbene **I** reacts with aldehyde **1** to afford Breslow intermediate **II**, which could lead to benzoin (**6**), or tetrahedral intermediate **III** when treated with α-imino ester **4**. Intermolecular proton transfer from **III** gives intermediate **IV**, which could release the product **5** and the carbene **I** to complete the catalytic system. We speculated that the desired product **5** is thermodynamically more stable than **6** and the formation of **5** is the irreversible step, from the finding that cross-coupled product **5** is predominantly obtained under the reaction conditions.

## Conclusion

In conclusion, we have developed a direct, atom-efficient synthesis of α-amino-β-keto esters by an umpolung reaction. We found that the NHC-catalyzed cross-aza-benzoin reaction of aldehydes with *N*-PMP-imino ester proceeds chemoselectively under very mild conditions. Therefore, the reaction is tolerant of a range of functional groups and substituents, including aliphatic aldehydes, and thus this method would be an attractive approach for deriving various α-amino-β-keto ester derivatives, which are useful synthetic blocks and valuable pharmaceutical intermediates.

## Supporting Information

File 1 Experimental details and characterization of the synthesized compounds.
