# Supplementary material for: N-Heterocyclic carbene-catalyzed direct cross-aza-benzoin reaction: Efficient synthesis of α-amino-β-keto esters
Source: Beilstein J Org Chem. 2012 Sep 10;8:1499–504. doi: 10.3762/bjoc.8.169 (PMC3458774; doi:10.3762/bjoc.8.169)
Supplement: File 1 — Experimental details and characterization of the synthesized compounds. [file Beilstein_J_Org_Chem-08-1499-s001.pdf]

Supporting Information

for

N-Heterocyclic carbene-catalyzed direct cross-aza-benzoin reaction: Efficient synthesis of α-amino-β-keto esters

Takuya Uno, Yusuke Kobayashi and Yoshiji Takemoto*

Address: Graduate School of Pharmaceutical Sciences, Kyoto University, Yoshida, Sakyo-ku, Kyoto 606-8501, Japan

Email: Yoshiji Takemoto - takemoto@pharm.kyoto-u.ac.jp

*Corresponding author

Experimental details and characterization of the synthesized compounds

Contents

General…… S2
Materials…… S2
Synthesis of catalysts…… S2
Synthesis of imino ester **4**…… S3
Typical procedure…… S3
Characterization data…… S3–7
Cross-over experiment…… S7
References…… S8

General

All reactions were carried out under a positive atmosphere of argon in dried glassware unless otherwise noted. Solvents were dried and distilled according to standard protocols. All melting points were measured on a Yamamoto micro melting point apparatus and are uncorrected. ^{1}H and ^{13}C NMR spectra were recorded in $CDCl_3$ at 500 MHz and at 126 MHz, respectively; tetramethylsilane (TMS) was used as an internal standard. IR spectra were recorded on a JASCO FT/IR-410 or FT/IR-4100 Fourier-transform infrared spectrometer. Low- and high-resolution mass spectra were recorded on a JEOL JMS-01SG-2 or JMS-HX/HX 110A mass spectrometer. Elemental analyses were performed on a YANACO CHN CORDER MT-6 spectrometer. Column chromatography was performed on Merck silica gel 60 (230–400 mesh). Reactions and chromatography fraction were analyzed by employing precoated silica gel plates (Merck Silica Gel F_{254}). Visualization was accomplished with UV light and/or staining with appropriate stains ($KMnO_4$, anisaldehyde, vaniline, ninhydrin or phosphomolybdic acid).

Materials

Unless otherwise noted, materials were purchased from Tokyo Kasei Co., Aldrich Inc., and other commercial suppliers, and were used without purification. Liquid aldehydes were distilled prior to use. Solid aldehydes were washed acid-free with 10% aq K_2CO_3 solution prior to use. For the cross coupling experiments, K_2CO_3 was finely ground prior to use.

Synthesis of catalysts

2-Phenyl-6,7-dihydro-5*H*-pyrrolo[2,1-*c*][1,2,4]triazol-2-ium tetrafluoroborate [1] **(3d)**

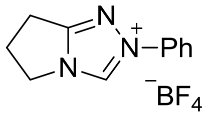

Based upon the literature procedure [1], trimethyloxonium tetrafluoroborate (4.47 g, 30 mmol) was added to a solution of pyrrolidin-2-one (2.55 g, 30 mmol) in CH_2Cl_2 (180 mL) and stirred at room temperature overnight. Phenylhydrazine (3.24 g, 30 mmol) was added and stirred for 24 hours before concentration in vacuo. The resulting crude residue was dissolved in MeOH (15 mL), chlorobenzene (20 mL), and triethylorthoformate (60 mL) and the mixture was refluxed overnight at 120 °C. The resultant solid was filtered and recrystallized from MeOH to afford **3d** (4.9 g, 60%) as a tan solid.

2-Pentafluorophenyl-6,7-dihydro-5*H*-pyrrolo[2,1-*c*][1,2,4]triazol-2-ium tetrafluoroborate [2] **(3e)**

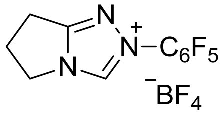

Following a modified procedure described in the literature [2], trimethyloxonium tetrafluoroborate (4.47 g, 30 mmol) was added to a solution of pyrrolidin-2-one (2.55 g, 30 mmol) in CH_2Cl_2 (180 mL) and the reaction mixture was stirred at room temperature overnight. Pentafluorophenylhydrazine (5.94 g, 30 mmol) was then added, and the whole was stirred for 24 hours before concentration in vacuo. The resulting crude was dissolved

in MeOH (15 mL), chlorobenzene (20 mL), and triethylorthoformate (60 mL) and the mixture was refluxed overnight at 120 °C. The resultant solid was filtered and recrystallized from MeOH to afford **3e** (5.6 g, 52%) as a tan solid.

Synthesis of imino ester 4

Ethyl *N*-(4-methoxyphenyl)iminoacetate [3] **(4)**

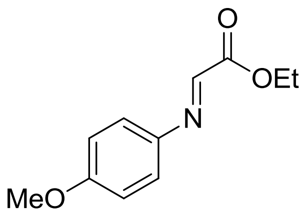

A mixture containing 1.52 g of ethyl glyoxylate (14.9 mmol), *p*-anisidine 1.84 g, 14.9 mmol), and anhydrous $MgSO_4$ (10 g) in toluene (30 mL) was stirred at room temperature for 30 min and the precipitate was then filtered. The filtrate was removed under reduced pressure to give the product **4** (3.1 g, 99%) as a yellow oil.

Typical procedure for cross-aza-benzoin reaction

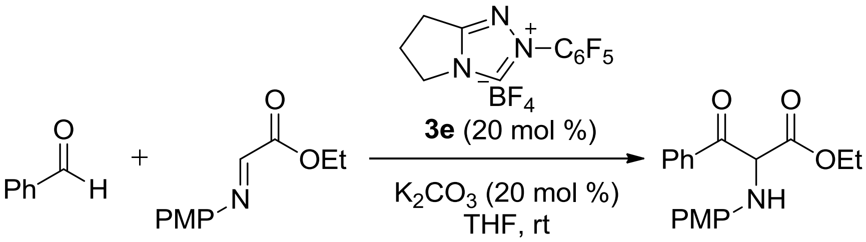

1a **4** **5a**

To a solution of iminoester **4** (80.8 mg, 0.33 mmol, 1.3 equiv) in THF (0.6 mL) were added NHC precatalyst **3e** (21.8 mg, 0.06 mmol, 20 mol %), aldehyde **1a** (0.3 mmol) and K_2CO_3 (8.1 mg, 0.06 mmol, 20 mol %) at room temperature. After being stirred at the same temperature for 24 hours, the reaction mixture was washed with aq $NaHSO_3$ solution and brine. The organic layer was dried over $MgSO_4$, and then concentrated in vacuo. The resulting residue was purified by silica gel column chromatography (hexane/ethyl ether 5:1) to give **5a** (65.8 mg, 70% yield).

Characterization data

Ethyl 2-[(4-methoxyphenyl)amino]-3-oxo-3-phenylpropanoate (5a)

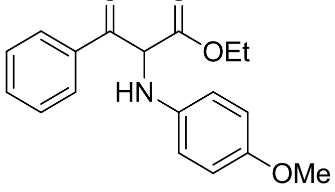

Yellow oil; IR (neat) 3400, 2984, 2936, 2905, 2833, 1748, 1640, 1572 cm^{-1}; ^1H NMR (500 MHz, $CDCl_3$) δ 8.12 (d, *J* = 7.2 Hz, 2H), 7.62 (t, *J* = 7.5 Hz, 1H), 7.50 (t, *J* = 7.8 Hz, 2H), 6.78 (d, *J* = 8.9 Hz, 2H), 6.72 (d, *J* = 8.9 Hz, 2H), 5.60 (s, 1H), 4.13 (q, *J* = 7.2 Hz, 2H), 3.73 (s, 3H), 1.09 (t, *J* = 7.2 Hz, 3H), one proton of NH was not recorded; ^{13}C NMR (126 MHz, $CDCl_3$) δ 192.4, 168.5, 153.0, 139.7, 134.1, 130.1, 128.9, 128.9, 115.3, 114.9, 64.5, 62.1, 55.6, 13.8; HRMS (FAB^+): Calcd for $C_{18}H_{19}NO_4$ (M^+) 313.1314; found: 313.1312.

Ethyl 2-[(4-methoxyphenyl)amino]-3-oxo-3-(4-chlorophenyl)propanoate (5b)

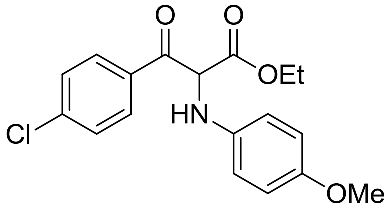

Yellow solid; mp 120–122 °C; IR (neat) 2990, 2914, 2911, 2844, 1750, 1652, 1577 cm^{-1}; ^1H NMR (500 MHz, $CDCl_3$) δ 8.06 (d, *J* = 8.9 Hz, 2H), 7.47 (d, *J* = 8.9 Hz, 2H), 6.79 (d, *J* = 8.9 Hz, 2H), 6.70 (d, *J* = 8.9 Hz, 2H), 5.52 (s, 1H), 4.14 (q, *J* = 5.8 Hz, 2H), 3.74 (s, 3H), 1.11 (t, *J* = 7.2 Hz, 3H), one proton of NH was not recorded; ^{13}C NMR (126 MHz, $CDCl_3$) δ 191.6, 168.5, 153.3, 141.0, 139.7, 132.7, 130.7, 129.3, 115.5, 115.1, 64.9, 62.5, 55.8, 14.1; Anal. Calcd for $C_{18}H_{18}ClNO_4$: C, 62.12; H, 5.22; N, 4.03; found: C, 61.90; H, 4.95; N, 4.02.

Ethyl 2-[(4-methoxyphenyl)amino]-3-oxo-3-(3-chlorophenyl)propanoate (5c)

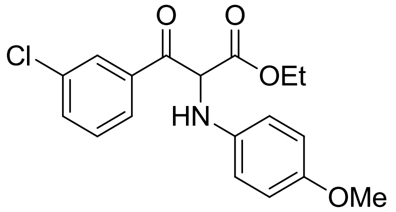

Yellow oil; IR (neat) 2985, 2922, 2889, 2840, 1752, 1655, 1560 cm^{-1}; ^1H NMR (500 MHz, $CDCl_3$) δ 7.94 (s, 1H), 7.83 (d, *J* = 7.8 Hz, 1H), 7.52 (d, *J* = 7.8 Hz, 1H), 7.27–7.22 (m, 1H), 6.79 (d, *J* = 8.9 Hz, 2H), 6.71 (d, *J* = 8.9 Hz, 2H), 5.56 (s, 1H), 4.14 (q, *J* = 7.2 Hz, 2H), 3.73 (s, 3H), 1.11 (t, *J* = 7.2 Hz, 3H), one proton of NH was not recorded; ^{13}C NMR (126 MHz, $CDCl_3$) δ 191.5, 168.4, 153.3, 141.1, 139.9, 132.2, 129.9, 129.7, 126.7, 125.7, 115.5, 115.0, 64.9, 62.5, 55.9, 14.1; HRMS (FAB^+): Calcd for $C_{18}H_{18}ClNO_4$ (M^+) 347.0924; found: 347.0921.

Ethyl 2-[(4-methoxyphenyl)amino]-3-oxo-3-(2-chlorophenyl)propanoate (5d)

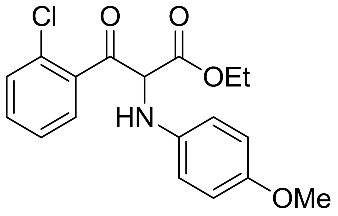

Yellow oil; IR (neat) 2991, 2934, 2891, 2824, 1754, 1649, 1570 cm^{-1}; ^1H NMR (500 MHz, $CDCl_3$) δ 8.06 (d, *J* = 8.9 Hz, 2H), 7.47 (d, *J* = 8.9 Hz, 2H), 6.78 (d, *J* = 8.9 Hz, 2H), 6.70 (d, *J* = 8.9 Hz, 2H), 5.59 (s, 1H), 4.15 (q, *J* = 7.2 Hz, 2H), 3.73 (s, 3H), 1.09 (t, *J* = 7.2 Hz, 3H), one proton of NH was not recorded; ^{13}C NMR (126 MHz, $CDCl_3$) δ 191.5, 168.4, 153.3, 141.1, 139.8, 132.0, 129.5, 129.2, 126.1, 125.9, 115.5, 115.0, 64.9, 62.5, 55.9, 14.1; HRMS (FAB^+): Calcd for $C_{18}H_{18}ClNO_4$ (M^+) 347.0924; found: 347.0921.

Ethyl 2-[(4-methoxyphenyl)amino]-3-oxo-3-(4-nitrophenyl)propanoate (5e)

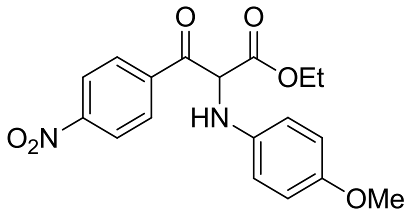

Orange oil; IR (neat) 2994, 2920, 2899, 2830, 1750, 1659, 1562 cm^{-1}; ^1H NMR (500 MHz, $CDCl_3$) δ 8.12 (d, *J* = 8.9 Hz, 2H), 7.52 (d, *J* = 8.9 Hz, 2H), 6.80 (d, *J* = 8.9 Hz, 2H), 6.72 (d, *J* = 8.9 Hz, 2H), 5.48 (s, 1H), 4.14 (q, *J* = 7.2 Hz, 2H), 3.72 (s, 3H), 1.11 (t, *J* = 7.2 Hz, 3H), one proton of NH was not recorded; ^{13}C NMR (126 MHz, $CDCl_3$) δ 192.5, 167.3, 153.0, 139.2, 137.9, 129.8,

123.7, 121.0, 115.3, 114.8, 64.7, 61.0, 55.6, 13.9; HRMS (FAB$^+$): Calcd for $C_{18}H_{18}N_2O_6$ (M^+) 358.1165; found: 358.1168.

Ethyl 2-[(4-methoxyphenyl)amino]-3-oxo-3-(4-cyanophenyl)propanoate (5f)

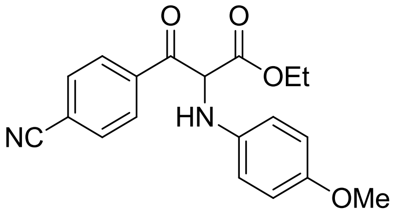

Orange oil; IR (neat) 2930, 2920, 2880, 2830, 1762, 1645, 1560 cm^{-1}; ^{1}H NMR (500 MHz, $CDCl_3$) δ 8.28 (d, *J* = 8.6 Hz, 2H), 7.62 (d, *J* = 8.6 Hz, 2H), 6.79 (d, *J* = 8.9 Hz, 2H), 6.69 (d, *J* = 8.9 Hz, 2H), 5.50 (s, 1H), 4.16 (q, *J* = 7.2 Hz, 2H), 3.76 (s, 3H), 1.13 (t, *J* = 7.2 Hz, 3H), one proton of NH was not recorded; ^{13}C NMR (126 MHz, $CDCl_3$) δ 192.1, 167.9, 152.6, 140.8, 132.5, 131.7, 129.5, 129.1, 115.4, 114.9, 114.6, 65.1, 62.6, 55.6, 13.9; HRMS (FAB$^+$): Calcd for $C_{19}H_{18}N_2O_4$ (M^+) 338.1267; found: 338.1265.

Methyl 4-{3-ethoxy-2-[(4-methoxyphenyl)amino]-3-oxopropanoyl}benzoate (5g)

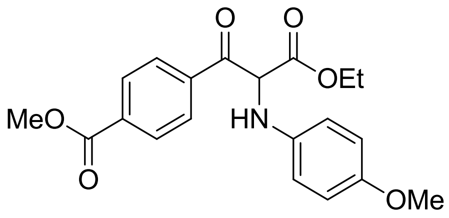

Yellow oil; IR (neat) 3005, 2910, 2909, 2820, 1755, 1630, 1579 cm^{-1}; ^{1}H NMR (500 MHz, $CDCl_3$) δ 8.17–8.11 (m, 4H), 6.78 (d, *J* = 8.9 Hz, 2H), 6.71 (d, *J* = 8.9 Hz, 2H), 5.58 (s, 1H), 4.14 (q, *J* = 7.2 Hz, 2H), 3.95 (s, 3H), 3.74 (s, 3H), 1.10 (t, *J* = 7.2 Hz, 3H), one proton of NH was not recorded; ^{13}C NMR (126 MHz, $CDCl_3$) δ 192.3, 168.1, 165.9, 153.1, 139.4, 137.4, 129.8, 123.7, 121.0, 115.3, 114.8, 64.9, 61.4, 55.6, 52.5, 13.9; HRMS (FAB$^+$): Calcd for $C_{20}H_{21}NO_6$ (M^+) 371.1369; found: 371.1370.

Ethyl 2-[(4-methoxyphenyl)amino]-3-oxo-3-(4-methylphenyl)propanoate (5h)

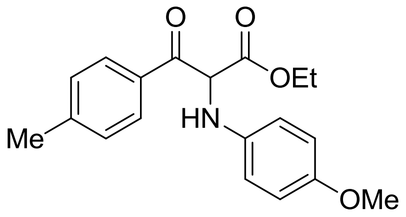

Yellow oil; IR (neat) 2990, 2912, 2901, 2802, 1752, 1655, 1559 cm^{-1}; ^{1}H NMR (500 MHz, $CDCl_3$) δ 8.02 (d, *J* = 8.3 Hz, 2H), 7.30 (d, 8.3 Hz, 2H), 6.77 (d, *J* = 8.9 Hz, 2H), 6.72 (d, *J* = 8.9 Hz , 2H), 5.57 (s, 1H), 4.12 (q, *J* = 7.2 Hz, 2H), 3.73 (s, 3H), 2.42 (s, 3H), 1.10 (t, *J* = 7.2 Hz, 3H), one proton of NH was not recorded; ^{13}C NMR (126 MHz, $CDCl_3$) δ 191.8, 168.6, 152.9, 145.3, 139.8, 130.3, 129.4, 129.2, 115.2, 114.8, 64.3, 62.0, 55.6, 21.7, 13.8; HRMS (FAB$^+$): Calcd for $C_{19}H_{21}NO_4$ (M^+) 327.1471; found: 327.1471.

Ethyl 2-[(4-methoxyphenyl)amino]-3-oxo-3-(4-methoxyphenyl)propanoate (5i)

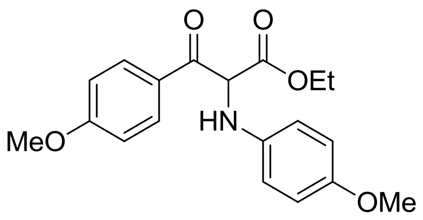

Yellow oil; IR (neat) 2988, 2901, 2899, 2849, 1752, 1645, 1561 cm^{-1}; ^{1}H NMR (500 MHz, $CDCl_3$) δ 8.11 (d, *J* = 10.7 Hz, 2H), 6.96 (d, *J* = 10.7 Hz, 2H), 6.80 (d, *J* = 8.9 Hz, 2H), 6.73 (d, *J* = 8.9 Hz, 2H), 5.55 (s, 1H), 4.12 (q, *J* = 7.2 Hz, 2H), 3.73 (s, 3H), 2.17 (s, 3H), 1.10 (t, *J* = 7.2 Hz, 3H), one proton of NH was not

recorded; ^{13}C NMR (126 MHz, $CDCl_3$) δ 190.5, 168.8, 164.3, 152.9, 139.8, 132.2, 131.6, 129.8, 115.2, 114.8, 64.1, 62.0, 55.6, 55.5, 13.9; HRMS (FAB$^+$): Calcd for $C_{19}H_{21}NO_5$ (M^+) 343.1420; found: 343.1422.

Ethyl 2-[(4-methoxyphenyl)amino]-3-oxo-3-(thiophen-3-yl)propanoate (5j)

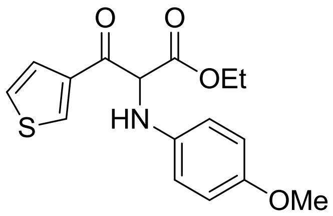

Orange oil; IR (neat); 2985, 2920, 2901, 2845, 1766, 1655, 1572 cm^{-1}; ^{1}H NMR (500 MHz, $CDCl_3$) δ 8.41 (dd, J_1 = 3.7 Hz, J_2 = 1.0 Hz, 1H), 7.66 (dd, J_1 = 5.0 Hz, J_2 = 1.0 Hz, 1H), 7.34 (dd, J_1 = 5.0 Hz, J_2 = 3.7 Hz, 1H), 6.79 (d, J = 8.8 Hz, 2H), 6.68 (d, J = 8.8 Hz, 2H), 5.38 (s, 1H), 4.16 (q, J = 7.3 Hz, 2H), 3.72 (s, 3H), 1.13 (t, J = 7.3 Hz, 3H), one proton of NH was not recorded; ^{13}C NMR (126 MHz, $CDCl_3$) δ 186.2, 168.6, 153.0, 139.6, 138.9, 134.6, 127.4, 126.5, 115.2, 114.9, 65.9, 62.2, 55.5, 14.8; HRMS (FAB$^+$): Calcd for $C_{16}H_{17}NO_4S$ (M^+) 319.0878; found: 319.0877.

Ethyl 2-[(4-methoxyphenyl)amino]-3-oxo-3-(furan-2-yl)propanoate (5k)

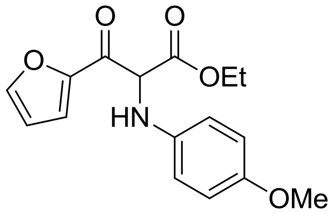

Yellow oil; IR (neat) 2950, 2294, 2860, 2811, 1720, 1661, 1565 cm^{-1}; ^{1}H NMR (500 MHz, $CDCl_3$) δ 7.68 (d, J = 1.7 Hz, 1H), 7.46 (d, J = 4.0 Hz, 1H), 6.79 (d, J = 8.9 Hz, 2H), 6.70 (d, J = 8.9 Hz, 2H), 6.60 (dd, J_1 = 4.0 Hz, J_2 = 1.7 Hz , 1H), 5.34 (s, 1H), 4.18 (q, J = 6.6 Hz, 2H), 3.74 (s, 3H), 1.16 (t, J = 7.2 Hz, 3H), one proton of NH was not recorded; ^{13}C NMR (126 MHz, $CDCl_3$) δ 181.4, 168.1, 153.1, 150.4, 147.7, 139.6, 120.0, 115.4, 114.8, 11.27, 63.5, 62.2, 55.6, 13.9; HRMS (FAB$^+$): Calcd for $C_{16}H_{17}NO_4$ (M^+) 303.1107; found: 303.1106.

Ethyl 2-[(4-methoxyphenyl)amino]-3-oxobutanoate (5l)

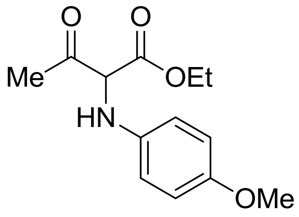

Yellow oil; IR (neat) 2953, 2884, 1655, 1600, 1501 cm^{-1}; ^{1}H NMR (500 MHz, $CDCl_3$) δ 6.80 (d, J = 8.8 Hz, 2H), 6.74 (d, J = 8.8 Hz, 2H), 5.72 (s, 1H), 4.25 (q, J = 7.2 Hz, 2H), 3.78 (s, 3H), 1.95 (s, 3H), 1.15 (t, J = 7.2 Hz, 3H), one proton of NH was not recorded; ^{13}C NMR (126 MHz, $CDCl_3$) δ 175.9, 162.5, 148.8, 129.6, 111.5, 110.6, 60.9, 59.7, 51.6, 21.5, 14.1; HRMS (FAB$^+$): Calcd for $C_{13}H_{17}NO_4$ (M^+) 251.1158; found: 251.1152.

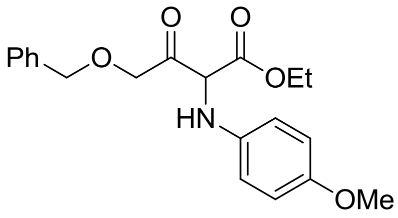

Ethyl 4-(benzyloxy)-2-[(4-methoxyphenyl)amino]-3-oxo-butanoate (5m)

Yellow oil; IR (neat) 2984, 2936, 2905, 1748, 1640, 1572 cm^{-1}; ^{1}H NMR (500 MHz, $CDCl_3$) δ 7.30–7.24 (m, 2H), 7.22–7.18 (m, 3H), 6.77 (d, J = 8.7 Hz, 2H), 6.71 (d, J = 8.7 Hz, 2H), 5.45 (s, 1H), 4.80 (s, 2H), 4.52 (s, 2H), 4.22 (q, J = 7.2 Hz, 2H), 3.80 (s, 3H),

1.15 (t, *J* = 7.2 Hz, 3H), one proton of NH was not recorded; ^{13}C NMR (126 MHz, $CDCl_3$) δ 200.9 168.5, 153.5 139.7, 137.9, 128.9, 128.6, 128.4, 115.3, 114.9, 74.8, 74.0, 64.5, 62.1, 55.6, 13.8; HRMS (FAB$^+$): Calcd for $C_{20}H_{23}NO_5$ (M^+) 357.1576; found: 357.1580.

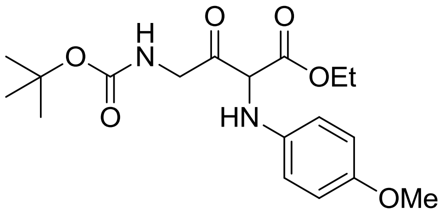

Ethyl 4-[(*tert*-butoxycarbonyl)amino]-2-[(4-methoxyphenyl)amino]-3-oxobutanoate (5n)
Yellow oil; IR (ATR) 2990, 2921, 2890, 1748, 1640, 1552 cm^{-1}; ^{1}H NMR (500 MHz, $CDCl_3$) δ 6.78 (d, *J* = 8.9 Hz, 2H), 6.69 (d, *J* = 8.9 Hz, 2H), 5.72 (s, 1H), 4.25 (q, *J* = 7.2 Hz, 2H), 4.12 (s, 2H), 3.78 (s, 3H), 1.39 (s, 9H), 1.15 (t, *J* = 7.2 Hz, 3H), two protons of NH were not recorded; ^{13}C NMR (126 MHz, $CDCl_3$) δ 199.6, 167.9, 156.0, 139.0, 130.5, 115.5, 115.2, 80.3, 64.5, 62.1, 55.6, 48.9, 28.4, 13.8; LRMS (FAB$^-$) 366 (M^+); HRMS (FAB$^-$): Calcd. for $C_{18}H_{26}N_2O_6$ (M^+) 366.1791; found: 366.1792.

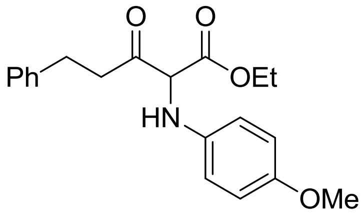

Ethyl 2-[(4-methoxyphenyl)amino]-3-oxo-5-phenylpentanoate (5o)
Yellow oil; IR (neat) 2984, 2936, 2905, 1748, 1640, 1572 cm^{-1}; ^{1}H NMR (500 MHz, $CDCl_3$) δ 7.30–7.26 (m, 2H), 7.22–7.18 (m, 3H), 6.77 (d, *J* = 8.6 Hz, 2H), 6.68 (d, *J* = 8.6 Hz, 2H), 5.45 (s, 1H), 4.25 (q, *J* = 7.2 Hz, 2H), 3.79 (s, 3H), 2.83 (m, 2H), 2.78 (m, 2H), 1.14 (t, *J* = 7.2 Hz, 3H), one proton of NH was not recorded; ^{13}C NMR (126 MHz, $CDCl_3$) δ 200.4, 168.5, 153.0, 139.7, 134.1, 130.1, 128.9, 128.9, 115.3, 114.9, 64.5, 62.1, 55.6, 35.5, 28.1, 13.8; HRMS (FAB$^+$): Calcd for $C_{20}H_{23}NO_4$ (M^+) 341.1627; found: 341.1625.

Cross-over experiment (Scheme 2)

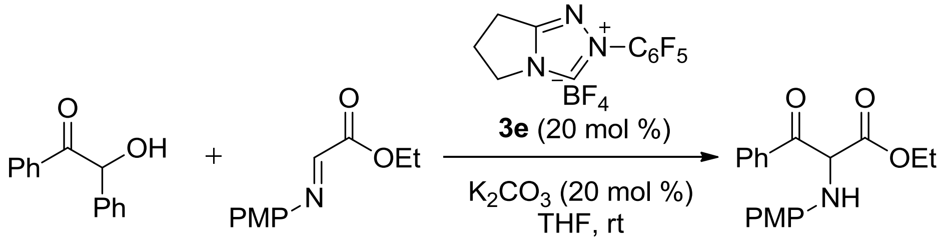

6 4 5a

To a solution of iminoester **4** (41.4 mg, 0.2 mmol) in THF (2.0 mL) were added NHC precatalyst **3e** (14.5 mg, 0.04 mmol, 20 mol %), benzoin (**6**, 21.2 mg, 0.1 mmol), and K_2CO_3 (5.5 mg, 0.04 mmol, 20 mol %) at room temperature. After being stirred at the same temperature for 24 hours, the reaction mixture was washed with aq $NaHSO_3$ solution and brine. The organic layer was dried over

$MgSO_4$, and then concentrated in vacuo. The resulting residue was purified by silica gel column chromatography (hexane/ethyl ether 5:1) to give **5a** (25.0 mg, 40% yield).

References

1. Thomson, J. E.; Rix, K.; Smith, A. D. *Org. Lett.* **2006,** *8*, 3785–3788. doi:10.1021/ol061380h
2. Kerr, M. S.; de Alaniz, J. R.; Rovis, T. *J. Org .Chem.* **2005,** *70,* 5725–5728. doi:10.1021/jo050645n
3. Borrione, E.; Prato, M.; Scorrano, G.; Stivanello, M.; Lucchini, V. *J. Heterocycl. Chem.* **1998,** *25,* 1831–1835. doi:10.1002/jhet.5570250644
